# Treatment of Patients with Distant Metastases from Phyllodes Tumor of the Breast

**DOI:** 10.1007/s00268-015-3262-7

**Published:** 2015-10-13

**Authors:** J. W. Mituś, P. Blecharz, T. Walasek, M. Reinfuss, J. Jakubowicz, J. Kulpa

**Affiliations:** Department of Surgical Oncology, Centre of Oncology, Maria Skłodowska-Curie Memorial Institute, Cracow Branch, ul. Garncarska 11, 31-115 Kraków, Poland; Department of Anatomy, Collegium Medicum, Jagiellonian University, ul. Kopernika 12, 31-034 Kraków, Poland; Department of Gynecologic Oncology, Centre of Oncology, Maria Skłodowska-Curie Memorial Institute, Cracow Branch, ul. Garncarska 11, 31-115 Kraków, Poland; Department of Radiotherapy, Centre of Oncology, Maria Skłodowska-Curie Memorial Institute, Cracow Branch, ul. Garncarska 11, 31-115 Kraków, Poland; Department of Clinical Oncology, Centre of Oncology, Maria Skłodowska-Curie Memorial Institute, Cracow Branch, ul. Garncarska 11, 31-115 Kraków, Poland; Department of Clinical Biochemistry, Centre of Oncology, Maria Skłodowska-Curie Memorial Institute, Cracow Branch, ul. Garncarska 11, 31-115 Kraków, Poland

## Abstract

**Background:**

Here, the treatment methods and results of patients with phyllodes tumor of the breast (PT) with distant metastases at a single institution are presented.

**Methods:**

A retrospective analysis was performed on a group of 295 patients with PT treated from 1952 to 2010.

**Results:**

Distant metastases developed in 37 (12.5 %) patients; 3/160 (1.9 %) patients had benign PT, 6/36 (16.7 %) were considered borderline, and 28/99 (28.3 %) had malignant PT. Most frequently, the metastases were located in the lungs; 28 (75.7 %), bone 7 (18.9 %), brain 4 (10.8 %), and liver 2 (5.4 %). Metastases occurred on overage 21 months (2–57) after surgery. Patients with lung metastases were generally treated with monochemotherapy or polychemotherapy. In one patient Testosterone and in two patients resection of metastases combined with Doxorubicin were used. Patients with bones or brain metastases were treated with palliative radiotherapy only or combined with Doxorubicin. The mean survival (MS) from diagnosis of distant metastases (DM) was 7 months (2–17). The longest mean survival in patients with bones metastases was 11.8 months, the worst survival was for patients with brain metastases—2.8 months. Hormone therapy appeared to have low efficacy (MS: 2 months) as well as monochemotherapy (MS: 3–5 months). Improved MS was obtained using Doxorubicin (7 months) and Doxorubicin with Cisplatin, Cyclophosphamide, or Ifosfamide (9 months).

**Conclusion:**

The prognosis of patients with DM from PT is poor. The role of surgery and irradiation of such patients is very limited. There appears to be no role for the use of hormone therapy. This study showed that polychemotherapy with Doxorubicin and Ifosfamide suggest that it might be more effective than once thought.

## Introduction

Phyllodes tumor (PT) is a rare fibro-epithelial neoplasm of the breast that accounts for 0.2–2 % of breast tumors in women [1–9]. PT is classified as benign or malignant; however, distinguishing those histotypes is difficult and based on semi-quantitative evaluation of the stromal component criteria including nuclear pleomorphism, mitotic rate, overgrowth, cellularity, and aspects of tumor margins [1, 2, 4, 7, 10].

The main cause of treatment failure in patients with PT of the breast is the dissemination of the neoplasm, which is diagnosed in 1–15 % of all patients [2, 11, 12]. The clinical behavior of a PT is unpredictable. Distant metastases (DM) occur mainly in malignant and borderline PT; however, metastatic spread in the benign type of PT has been described [11, 12]. The incidence of metastatic disease among patients with malignant PT is estimated to be approximately 20–25 % [2, 3].

A metastatic PT carries a poor prognosis with an average survival time of less than 2 years [2, 12–14]. The treatment of DM in patients with PT is a matter of debate. The role of surgery and radiotherapy is limited, and there is no role for the use of hormone therapy [2, 6, 8, 15]. Chemotherapy has been used for many years; however, it is difficult to assess the real value of this treatment due to the small size of the study groups and the diversity of drugs used for treatment, as well as the doses and regimens applied [2, 6, 9, 15–18].

The aim of this study was to present the methods used and clinical outcomes of patients with DM from PT after treatment at a single institution in Poland.

## Materials and methods

Between January 1952 and February 2010, 295 women with PT of the breast were treated surgically at the Maria Skłodowska-Curie Memorial Institute of Oncology, Cancer Center in Cracow, Poland. All clinical characteristics, treatment options, and therapy outcomes were acquired by the review of patients’ charts. The diagnosis and specific microscopic type of neoplasm were confirmed according to the WHO classification. All histology slides were re-examined, and the diagnosis of histotypes of PT was based on World Health Organization criteria.

The five-year survival with no evidence of disease (NED) was used as the endpoint for the analysis. 5-year NED was chosen as the primary endpoint because it reflects distant tumor control better compared to other commonly used outcome measures. All patients were followed up for at least 5 years (or until death). The mean follow-up time was 11 years. The log-rank test was used for evaluation of significance. The level of statistical significance was set at *p* ≤ 0.05. A multivariate Cox proportional hazard model was applied to analyze influence of evaluated factors on the adjusted survival.

## Results

Among all 295 patients, 256 (86.8 %) survived the 5-year NED. Table 1 shows the clinical outcomes according to the PT histotypes.

**Table 1:** Results of the treatment in 295 patients with PT

Phyllodes tumor	No. of patients	5-year NED		Distant Metastases	
		No of patients	%	No. of patients	%
Benign	160	155	96.9	3^a^	1.9
Borderline	36	30	83.3	6	16.7
Malignant	99	71	71.7	28	28.3
Total	295	256	86.8	37^a^	12.5

^a^Two additional patients died without PT recurrences

The 5-year NED survival was observed in 96.9 % patients with benign PT, 83.3 % with borderline PT, and 71.7 % with malignant PT. Two patients in the group with benign PT died due to other reasons (myocardial infarct and cerebral hemorrhage). All other patients (3 benign, 6 borderline, and 28 malignant PT) who died during the 5-year follow-up developed DM.

Among 196 patients with benign and borderline PT, 9 (4.6 %) and among 99 patients with malignant PT, 28 (28.3 %) developed DM. The difference was statistically significant (log-rank test, *p* < 0.01).

This study focused on a group of 37 patients with distant metastases (DM) from PT. The median age of those patients was 54 (range 48–76 years of age). The tumor size, defined as the maximal dimension of the tumor, as reported in the pathology report, ranged from 4 to 8 cm (mean 6 cm, median 5.5 cm). The tumor measurements were as follows: <5 cm in 21 (56.8 %) patients, 5–7 cm in 13 (35.1 %) patients, and over 7 cm in 3 (8.1 %) patients. In two patients, regional lymph node metastases were also diagnosed. Table 2 shows the localization of DM.

**Table 2:** Localization of DM in the group of 37 patients with PT

Localization	No. of patients	%
Lung	24	64.9
Lung + bones	2	5.4
Lung + liver	2	5.4
Bones	5	13.5
Brain	4	10.9
Total	37	100.0

In the group studied, the most frequent metastases were to the lungs 28 (75.7 %). Metastases also occurred to bones 7 (18.9 %), brain 4 (10.8 %), and liver 2 (5.4 %). DM were observed, on average, 21 months (range 2–57) after surgery. Among those patients that developed DM, 91.9 % (34/37) demonstrated DM within 3 years of initial treatment.

Table 3 shows the methods and clinical outcomes of treatment in 37 patients with phyllodes tumor DM.

**Table 3:** Methods and results of the treatment in 37 patients with phyllodes tumor DM

Localization of DM	Treatment	No. of patients	Results	
			Remission	Survival (months) from diagnosis of DM
Lung (24 patients)	Testosterone	1	NM^a^	2
	Cyclophosphamide	2	NM	3 and 3
	Methotrexate + dactinomycin + Cyclophosphamide + vincristine	3	NM	4, 4, and 4
	Ifosfamide	4	PR—1 patient NR—3 patients	6 6, 5, and 5
	Doxorubicin	6	PR—4 patients NR—2 patients	9, 9, 8, and 5 6 and 5
	Resection of individual metastases + Doxorubicin	2	CR	8 and 9
	Doxorubicin + Cisplatin	3	PR—3 patients	9, 8, and 9
	Doxorubicin + Ifosfamide	3	CR—1 patients PR—2 patients	11 10 and 9
Lung + liver (2 patients)	Doxorubicin + Cisplatin	2	PR—1 patient NR—1 patient	8 6
Lung + bones (2 patients)	Doxorubicin + CyclophosphamideDoxorubicine + palliative radiotherapy	2	PR—1 patient NR—1 patient	9 9
Bones (5 patients)	Doxorubicin + palliative radiotherapy	5	PR—5 patients	11, 11, 10, 10, and 17
Brain (4 patients)	Palliative radiotherapy Palliative radiotherapy + Doxorubicin	3 1	NR NR	2, 3, and 3 3

*NR* no remission

*PR* partial remission

*CR* complete remission

Between 1952 and 1985, six patients with lung metastases were treated with hormone therapy (Testosterone), monochemotherapy (Cyclophosphamide), and polychemotherapy (Methotrexate + Dactinomycin + Cyclophosphamide + Vincristine).

Between 1986 and 2010, 22 patients with lung metastases were treated as follows:

- Ten patients with monochemotherapy: Ifosfamide (four patients) or Doxorubicin (six patients)
- Ten patients with polychemotherapy: Doxorubicin + Cisplatin (five patients), Doxorubicin + Ifosfamide (three patients), Doxorubicin + Cyclophosphamide (two patients)
- Two patients: resection of metastases was combined with Doxorubicin.

In 11 patients with bone or brain metastases, palliative radiotherapy (PR) (20 Gy in five fractions) was used (two patients with lung and bone metastases: PR was combined with Doxorubicin + Cyclophosphamide, five patients with bone metastases only received PR + Doxorubicin, one patient with brain metastases received PR + Doxorubicin, and three patients with brain metastases received PR only).

The mean survival (MS) from the diagnosis of distant metastases in the study patients was 7 months (2–17), and the median 8 months. Among 24 patients with lung metastases: complete remission (CR) occurred in three patients (MS 9.1 months), partial remission (PR) in 10 patients (MS 8.2 months), and no remission (NR) in 11 patients (MS 4.3 months). The MS of patients with lung and liver metastases was 7 months, and with lung and bone metastases it was 9 months. In the group studied here, two patients were treated with resection of individual lung metastasis and Doxorubicin; they survived 8 and 9 months after treatment. The longest MS was among patients with bone metastases (11.8 months), and the worst MS was among patients with brain metastases (2.8 months).

Table 3 shows the low efficacy of hormone therapy (MS 2 months) and monochemotherapy (Cyclophosphamide MS 3 months, Ifosfamide 5 months). A longer MS was obtained after the use of Doxorubicin monochemotherapy (7 months), and Doxorubicin polychemotherapy with Cisplatin, Cyclophosphamide, or Ifosfamide (9 months).

## Discussion

For the study group of 295 patients with PT, 37 (12.5 %) presented with DM, consistent with prior literature [1–3, 5, 6, 9, 11, 12, 14, 19–22]. Among 160 patients with benign PT, 3 (1.9 %) developed DM, among 36 patients with borderline PT, 6 (16.7 %) and among 99 patients with malignant PT, 28 developed DM (28.3 %). The accepted frequency of DM in benign, borderline, and malignant PT is 0–2, 3–11, and 6–47 %, respectively [1–3, 5, 6, 9, 11–15, 22–24].

Most frequently, metastases are to the lung, bones, brain, and liver; this is consistent with the patients reported here [1, 2, 6, 11, 12]. DM from PT may also occur in other organs, such as the heart, pleura, oral cavity, larynx, nasal cavity, salivary glands, thyroid, adrenal glands, spleen, pancreas, kidney, stomach, small and large intestine, seminal glands, prostate, skin, ovary, and vulva to name some [5, 8, 9, 11, 25].

The time span between treatment of the primary tumor and possible manifestation of distant metastases varies greatly, and ranges from 1 month to over 10 years; however, the majority of DM of PT are noted within the first 3 years [2, 6, 12, 14, 20, 26]. In this study group of patients, DM were observed on average after 21 months (2-57), in other reports: 15 months (Abdala and Sakr), 14 months (Barrio et al.), 21 months (Kapiris et al.), 30 months (Fou et al.), and 53 months (Asoglu et al.) [6, 14, 20, 24, 26].

The role of surgery for the treatment of disseminated PT is limited to palliative management such as resection of individual metastases [2]. An effective method of palliative treatment may be irradiation of metastases, such as those to the bones, brain, and mediastinal lymph nodes. Five of the patients in this study, with bone metastases, had the longest mean survival of all patients with DM of PT (11.8 months) [2, 6, 19].

While the epithelial component of most PT contains estrogen and progesterone receptors, hormone therapy did not demonstrate any significant efficacy in the treatment of DM [4, 6, 8, 11].

In the patients with DM from a PT, chemotherapy has been considered the first-line treatment for many years [2, 9, 11, 15, 16, 25]. Many investigators have confirmed a low efficacy of monochemotherapy (Cyclophosphamide, Ifosfamide, Doxorubicin), although there are single reports of spectacular, usually short-lasting, regressions [2, 15, 16, 18, 25, 27]. Slightly better regression, although usually neither substantial nor long-lasting, is obtained after treatment with polychemotherapy (Doxorubicin with Cisplatin or Ifosfamide, Cisplatin with Etoposide [2, 15, 16, 18, 27]. Total regression of metastatic foci has been reported as well [2, 16, 17, 28].

In this study group of patients with lung metastases, there was a low efficacy of treatment with monochemotherapy with Cyclophosphamide (MS 3 months) or Ifosfamide (MS 5 months). There was a slightly better MS with monochemotherapy using Doxorubicin (MS 7 months) and Doxorubicin used in polychemotherapy with Cisplatin, Cyclophosphamide, or Ifosfamide (MS months). The best survival was observed in patients with bone metastases only that were treated with palliative radiotherapy and Doxorubicin (MS 11.8 months). No evident neurological improvement was noted in the four patients with brain metastases treated with palliative radiotherapy (MS 2.8 months).

Investigators differ in their assessment of survival in patients with PT once manifestations of DM are noted. Reinfuss et al. reported an MS of 4 months, Abdalla and Sakr 5 months (1–11), Barth et al. 12 months, Kapiris et al. 16.6 months (1–24), and de Ross et al. 17 months (12–15) [6, 12–14, 29]. It is generally reported that the survival time of patients with DM from PT is shorter than 24 months, even though there are longer survival times, even up to 16 years [2, 18]. In this group of patients, the MS was 7 months (2–17) and the median was 8 months.

Phyllodes tumor DM carries a poor prognosis. In the 1950s and 1970s, chemotherapy was shown to have minimal impact on survival of patients. However, data from the literature and the results of this study with polychemotherapy (Doxorubicin + Ifosfamide or Cisplatin) suggest that chemotherapy may be more efficient than previously thought [2, 7, 15, 16]. The NCCN recommends that treatment of DM in patients with PT follow the algorithm outlined in the NCCN Soft Tissue Sarcoma Clinical Practice Guidelines in Oncology and novel drugs (Docetaxel + Gemcitabine, Pazopanib, or Trabectedin) may be used for example [30]. Future studies to identify relevant molecular targets should be carried out in order to define more effective therapies for DM in patients with PT. In 2013, Jardim et al. described for the first time an NRAS mutation with concomitant activation of P13 K/Akt/mTOR with PT. The investigators reported markers for sensitivity to taxane-based therapies, especially albumin-bound paclitaxel [31].

## Conclusion

In conclusion, on the basis of the results of this study and data from the literature, the prognosis of patients with DM from PT is poor, with an average survival of less than 2 years after diagnosis. The role of surgery and irradiation in the treatment of these patients is very limited (resection of individual DM, palliative irradiation DM in bones or brain), and there is no role for hormone therapy. Our findings of multidrug systemic therapy with Doxorubicin and Ifosfamide suggest that chemotherapy may be more efficient than once thought. At present, patients with DM from PT of the breast should be treated in the same manner as are patients with metastatic soft tissue sarcoma.
